# Equity-centred, nurse-led implementation of artificial intelligence in community maternal and child health nursing: a conceptual framework for low-resource settings

**DOI:** 10.3389/fpubh.2026.1879677

**Published:** 2026-07-15

**Authors:** Manar Fayez Alruwaili, Ugwu Okechukwu Paul-Chima

**Affiliations:** 1 Department of Maternal and Child Health Nursing, College of Nursing, Jouf University, Sakaka, Saudi Arabia; 2 Department of Research and Publication, Kampala International University, Kampala, Uganda

**Keywords:** algorithmic bias, artificial intelligence, digital health, health equity, humanised care, implementation science, low-resource settings, maternal and child health nursing

## Abstract

Substantial yet preventable maternal and neonatal mortality persists across low- and middle-income countries (LMICs), where shortages of skilled maternal and child health (MCH) nurses, inadequate infrastructure and socioeconomic inequalities continue to limit access to quality care. Artificial intelligence (AI), particularly machine-learning-driven decision-support and predictive systems, is increasingly proposed to strengthen MCH services through risk stratification, early diagnosis and clinical decision-making. However, evidence supporting its equitable, contextually appropriate and sustainable implementation in resource-constrained settings remains limited. Existing literature has inadequately addressed the structural, ethical and relational dimensions of AI adoption from a nursing perspective, and there is a lack of frameworks integrating equity, nurse-led governance and contextual adaptation for LMICs. Drawing on a purposive narrative review, this Perspective proposes an equity-centred, nurse-led framework for AI-assisted MCH nursing in low-resource settings. The framework comprises six interdependent domains: contextual algorithm validation, humanised and relational nursing care, community co-design and engagement, digital equity and inclusion, transparent data governance, and longitudinal monitoring and evaluation. Its novelty lies in positioning equity as a prerequisite for deployment, identifying the nurse–patient relationship as the principal mechanism through which AI influences outcomes, and assigning governance authority to nurses and communities. A phased implementation pathway is proposed to guide responsible deployment. The framework remains conceptual and requires prospective empirical validation. Responsible AI integration in MCH nursing demands equity-centred governance, investment in nursing capacity, community participation and locally grounded validation strategies.

## Introduction

Maternal and child health (MCH) remains a central global public health priority. Despite substantial reductions in maternal and under-five mortality over recent decades, preventable deaths remain heavily concentrated in low- and middle-income countries (LMICs). Current global estimates indicate that approximately 287,000 maternal deaths occur annually, with Sub-Saharan Africa and South Asia accounting for more than 85% of this burden (1). The major direct causes haemorrhage, sepsis, pre-eclampsia, unsafe abortion and obstructed labour are largely preventable with timely skilled care and effective referral systems. Neonatal mortality similarly remains unacceptably high, with an estimated 2.3 million neonatal deaths recorded in 2022, predominantly occurring in LMICs within the first hours and days of life (1). Childhood immunisation inequities compound this burden: an estimated 20.5 million children were zero-dose or under-vaccinated in 2023, concentrated in communities affected by poverty, conflict, displacement and fragile health systems (2).

Community MCH nurses are indispensable to national health systems, particularly in LMICs where they often provide the primary interface between communities and health services. They deliver antenatal care, support safe delivery, conduct neonatal assessments, provide breastfeeding counselling, administer immunisations, deliver health education and facilitate early referral. Yet nursing workforce shortages are acute: the World Health Organization (WHO) has estimated a global shortfall of millions of nurses, concentrated predominantly in LMICs and disproportionately affecting MCH services in rural and underserved communities (3). Community nursing practice in these settings is characterised by limited supervision, inadequate supplies, fragmented health information systems and constrained opportunities for continuing professional development.

Artificial intelligence (AI) has emerged as a visible component of health system transformation globally. For the purposes of this Perspective, AI-assisted MCH nursing is defined narrowly as the application of machine-learning and related data-driven predictive or decision-support systems—including risk-prediction models, automated triage and algorithmic clinical decision support—to inform nursing assessment, risk stratification and referral. This definition deliberately distinguishes AI from the broader family of digital health interventions, such as short-message reminders, interactive voice response, static educational content and routine telehealth, which may incorporate AI but frequently do not. The distinction matters because predictive and decision-support algorithms carry specific technical characteristics, evidence requirements, failure modes and governance obligations notably algorithmic bias, dataset dependence and clinical accountability that differ materially from those of general digital health tools (4–7). Conflating the two categories has been a recurring weakness in the literature and risks transferring evidence of effectiveness from one to the other without justification (8–11).

In MCH specifically, AI-based predictive and decision-support tools have been explored to anticipate obstetric complications, support risk stratification, identify neonatal sepsis earlier and assist nurse-led triage where specialist oversight is scarce (12–14). Several LMIC-focused initiatives illustrate the exploratory potential of this integration, though the strength of available evidence varies considerably across contexts and most reports are at a feasibility or pilot stage (12). AI-assisted risk-stratification approaches have been described in sub-Saharan African and South Asian settings to support nurse-led identification of high-risk pregnancies, but peer-reviewed evidence on real-world effectiveness in these contexts remains preliminary, and external validity and scalability have not yet been established (12). These examples are presented as illustrations of implementation contexts and technological possibilities rather than as demonstrations of proven effectiveness at scale, and further rigorous, AI-specific evaluation is required.

Nevertheless, the integration of AI into MCH nursing raises fundamental concerns that the current literature has not adequately resolved. First, many AI models have been trained on datasets from high-income countries or narrow demographic groups, limiting their validity in LMIC populations where disease patterns, comorbidities and health-seeking behaviour differ substantially (12, 15). Second, AI tools may widen disparities when communities lack reliable electricity, internet connectivity, smartphones, digital literacy or trust in technology-mediated services (16). Third, the risk of eroding humanised care is real: over-reliance on automated triage or predictive algorithms may reduce meaningful nurse–patient interaction in contexts where trust, empathy and cultural sensitivity are fundamental to care-seeking behaviour. Fourth, governance frameworks in many LMICs remain insufficient to ensure accountability, informed consent, data protection and algorithmic transparency (4, 10).

The central challenge is therefore not whether AI can improve efficiency in MCH services, but how it can be implemented in ways that are contextually valid, equitable, nurse-led and ethically governed. Current literature on AI in MCH nursing has focused predominantly on technical performance metrics and short-term clinical outcomes, without adequately addressing implementation science dimensions, nursing workforce implications, equity effects or governance requirements. This Perspective aims to address this gap by: (i) critically appraising evidence on AI applications in MCH nursing; (ii) examining the risks of algorithmic bias, digital exclusion and erosion of humanised care; and (iii) proposing a conceptual framework for equity-centred, nurse-led AI implementation in low-resource settings.

As a Perspective, this article is based on a purposive, narrative review of the literature rather than a systematic review, and its conclusions should be read as drawing on selected evidence rather than on an exhaustive synthesis. The approach to identifying and selecting sources is described in full in the “Derivation of the framework” section. We searched PubMed, Scopus and Google Scholar, together with the publications of the WHO and allied agencies, between 2014 and 2026, using combinations of terms relating to artificial intelligence, machine learning, maternal and child health, nursing, health equity, algorithmic bias, implementation science and low-resource settings. Sources were selected purposively for their relevance to the four bodies of knowledge underpinning the framework, with priority given to peer-reviewed studies, systematic reviews and authoritative guidance; we did not apply formal inclusion criteria, quality appraisal or quantitative pooling, and the evidence cited is therefore illustrative of the field rather than a complete account of it. Throughout, statements about the evidence base should be understood within these limits.

## Artificial intelligence applications in maternal and child health nursing

AI-based predictive and decision-support tools are being applied across several domains of MCH care, with a growing but unevenly evaluated body of evidence from both high-income and LMIC settings. Machine-learning models can analyse maternal demographic, clinical and behavioural data to support prediction of obstetric complications, including pre-eclampsia, gestational diabetes mellitus, preterm birth and postpartum haemorrhage (12, 13). The evidence base for these specific applications is still maturing: a 2023 systematic review protocol set out a methodology for appraising machine-learning models for pre-eclampsia prediction but, as a protocol, reported no pooled findings (17). A subsequently published systematic review and meta-analysis of machine-learning models for pre-eclampsia prediction reported substantial heterogeneity across studies and a marked gap between internal model performance and external transferability, with limited validation in diverse populations (13). These findings underscore that strong internal discrimination in development cohorts should not be assumed to generalise to LMIC primary-care populations.

In neonatal and paediatric care, AI has been applied to neonatal sepsis prediction, interpretation of newborn vital signs and early-warning systems for clinical deterioration (14). A 2023 systematic review of machine-learning applications for neonatal sepsis found promising early results but noted that most models lacked prospective validation and that evidence from LMICs was sparse (14). For community nurses, AI-enabled decision-support systems may, in principle, improve triage consistency, risk-stratification reliability and referral appropriateness during home visits and outreach; however, robust evidence that such tools improve nurse-led decision-making in low-resource MCH settings remains limited and is a priority for future evaluation.

It is important to distinguish these AI-specific tools from the wider digital health interventions mHealth messaging, interactive voice response, appointment reminders and routine telehealth for which a larger effectiveness literature exists in LMICs (18, 19). Such interventions have improved maternal knowledge, breastfeeding practices and care-seeking in several settings, but this evidence pertains to communication and service-delivery tools rather than to predictive algorithms, and should not be read as evidence for the clinical effectiveness of AI decision-support. Telehealth experience during the COVID-19 pandemic similarly demonstrated that remote contact could sustain continuity of antenatal and postnatal care while also exposing risks of unequal access and reduced interpersonal contact, particularly among women from lower socioeconomic groups (19) a cautionary precedent for AI deployment, but not a substitute for AI-specific evidence.

AI should therefore not be treated as a stand-alone solution to MCH challenges. Many AI tools have been evaluated only through short-term pilot studies that provide limited evidence on sustained effectiveness, cost-effectiveness or equity effects (20). External validation in diverse LMIC populations is infrequently reported, and few studies include equity-stratified analyses or examine outcomes for the most marginalised groups. Crucially, AI does not address the structural determinants of MCH outcomes poverty, gender inequality, food insecurity, transport barriers, social stigma and fragile health systems without which technology-driven approaches are unlikely to produce sustained population-level improvements (21, 22).

## Humanised nursing care in the era of digital health

MCH nursing encompasses dimensions that extend far beyond clinical assessment, documentation and treatment. Pregnancy, childbirth, postpartum recovery and early childhood are emotionally and socially significant periods during which women may experience fear, grief, social vulnerability, stigma, trauma or intimate partner violence. Effective MCH nursing practice depends on empathy, respectful communication, continuity of relationships, cultural competence and psychosocial support. These dimensions constitute what is broadly understood as humanised care: an approach that recognises patients as persons with emotional, social and cultural needs rather than as data points within predictive models.

The concept of humanised care in MCH has been operationalised most comprehensively in the respectful maternity care framework, which emphasises freedom from harm and mistreatment, the right to information and informed consent, equitable care free of discrimination, and support for companionship and emotional needs during childbirth (23). A landmark global mixed-methods review documented that mistreatment of women during facility-based childbirth including physical abuse, disrespectful communication, lack of informed consent and discrimination is widespread and is a significant driver of delayed or avoided care-seeking (23). Culturally responsive and trauma-informed approaches are therefore essential complements to any technology-assisted care model.

Excessive reliance on automated triage or algorithmic decision support risks depersonalising care by reducing the time, attention and relational presence that nurses offer. This risk is particularly salient in MCH settings where delayed care-seeking is influenced by fear of mistreatment, social stigma, cultural beliefs or previous negative health system experiences. A practical tension arises: AI tools designed to improve efficiency may, if poorly integrated, undermine the nurse–patient relationship that determines whether women engage with antenatal services, accept referrals or maintain postnatal follow-up. Community resistance to digital interventions perceived as externally imposed, culturally inappropriate or as substitutes for human contact has been documented in several LMIC settings (22).

Nurses must therefore remain essential and empowered intermediaries between AI technologies and patients. AI should support professional judgement, clinical reasoning and relational continuity rather than replace these capacities. Nurse education and training should address not only digital competencies and AI literacy but also the ethical dimensions of AI use, including recognition of algorithmic limitations, protection of patient autonomy, commitment to informed consent, and responsibility for interpreting AI-generated recommendations within their broader clinical and social context (4, 24). Training should explicitly reinforce that AI-based tools are aids to clinical reasoning, not replacements for contextual judgement.

## Digital inequality and algorithmic bias in low-resource settings

The reliability and equity of AI systems depend heavily on the datasets used for their development and validation. Algorithmic bias in health AI systems arises through multiple, often interacting mechanisms. Selection bias occurs when training datasets systematically exclude or underrepresent specific population groups; measurement bias arises when outcome variables or predictor measures are defined or recorded inconsistently across groups; deployment bias occurs when a model is applied in a context that differs substantially from its development setting; and language bias affects natural language processing tools trained on data from dominant languages that inadequately represent linguistic diversity (15, 25). Each of these mechanisms is particularly consequential in MCH contexts in LMICs (11).

Maternal mortality and neonatal complications are disproportionately concentrated among populations that are simultaneously among the most underrepresented in digital health datasets: rural women, adolescent mothers, displaced and refugee populations, ethnic and linguistic minorities, and households living in extreme poverty (1, 21). An algorithm trained predominantly on data from urban tertiary hospitals in high-income countries may perform poorly when applied to predict complications in rural primary care settings in Sub-Saharan Africa. When such systems are deployed without local validation, they may generate systematically biased outputs, expose vulnerable populations to inappropriate or delayed care, and reinforce rather than reduce existing inequities. The investigation by Obermeyer et al. (25), which demonstrated that a widely used health algorithm assigned lower risk scores to Black patients despite equivalent disease severity, provides a cautionary example of how bias embedded in training data can produce discriminatory clinical outcomes.

Digital inequality compounds these concerns. In 2023, the International Telecommunication Union estimated that approximately 2.6 billion people remained unconnected to the internet, with the digital divide disproportionately affecting LMICs, rural communities and women (16). Many MCH contexts lack stable connectivity, reliable electricity supply, affordable smartphones and adequate digital literacy. Women in low-income settings face additional gender-based barriers to digital access: limited personal phone ownership, financial dependence on male household members, social norms restricting independent technology use and, in some contexts, concerns about surveillance through personal devices (26). Older caregivers and certain marginalised communities may face further exclusion.

Privacy and data governance concerns are equally salient. MCH data are highly sensitive, encompassing reproductive health history, antenatal risk assessments, neonatal outcomes, immunisation records and, in some contexts, information about intimate partner violence or social vulnerability. Weak regulatory frameworks in many LMICs may increase the risk of confidentiality breaches, unauthorised commercial data use, discriminatory data practices or politically motivated access to health records. Ethical AI implementation in MCH requires transparent governance structures that include informed consent procedures respecting community norms around data sharing, data minimisation principles, secure storage and transmission, meaningful accountability mechanisms and community-level trust-building processes (4, 27).

## From technological innovation to equitable, nurse-led digital health systems

Technological innovation alone cannot resolve the structural inequities that shape MCH outcomes in low-resource settings. AI-assisted care requires embedding within broader equity-oriented public health frameworks that prioritise access, accountability, cultural responsiveness and health system strengthening. Community MCH nurses are uniquely positioned to guide contextually sensitive implementation because they possess granular understanding of local barriers to care, family decision-making dynamics, cultural practices, community trust networks and the practical realities of resource-constrained clinical environments.

Implementation science frameworks offer important guidance for responsible AI integration. The NASSS (Non-adoption, Abandonment, Scale-up, Spread and Sustainability) framework highlights that health technology failure frequently results not from technical limitations but from inadequate attention to organisational context, workforce capacity, institutional mechanisms and the sociocultural embeddedness of care practices (20). Applying this framework to AI in MCH nursing suggests that implementation strategies must address workflow integration, community acceptability, nurse agency and system readiness alongside technical validation. Operationalising any of these domains requires financial, workforce and infrastructure investment that varies considerably across settings. Local algorithm validation demands trained data scientists, representative population datasets and iterative recalibration cycles, all of which entail sustained funding. Nurse training in AI literacy requires integration into pre-service curricula and continuing professional development. Community co-design demands time, facilitation capacity and translation resources that extend beyond token consultation. Governance frameworks require regulatory infrastructure that many health systems are still building. These requirements are substantial, and implementation strategies must be sequenced realistically within existing health system strengthening investments rather than treated as cost-neutral additions to existing programmes.

Future digital health strategies should adopt participatory, nurse-led implementation models that position nurses as active co-designers rather than passive recipients of externally developed technologies. Nurses should contribute systematically to AI system design, workflow integration, community engagement, policy development and outcome evaluation. Community participation is equally essential to ensure that AI-based interventions reflect local priorities, languages, health beliefs and expectations of respectful care (22). Co-design processes should be linguistically accessible and should prioritise the involvement of women who have used or are likely to use MCH services, including those from marginalised communities at greatest risk of digital exclusion.

Digital literacy strengthening should target both health workers and community members. Nurse training should encompass safe use of AI tools, recognition of algorithmic limitations, ethical decision-making in AI-mediated care and skills for interpreting AI-generated recommendations within clinical context. Community education should build confidence in using digital health platforms while ensuring that women who lack personal devices, connectivity or digital literacy are not excluded from essential care, including through community health worker intermediary models and shared device programmes. Infrastructure investment remains foundational: AI-assisted systems cannot operate effectively or equitably without reliable electricity, internet access, interoperable health information systems, secure data infrastructure and adequately trained staff. Governments, health institutions and development partners should resist technology-centred approaches that divert resources from foundational health system needs, and should instead ensure that AI adoption is sequenced appropriately within broader health system strengthening investments (21, 28).

## A conceptual framework for equity-centred, nurse-led AI-assisted MCH nursing

### Derivation of the framework

The framework was developed through a structured conceptual synthesis rather than a systematic review, and the method is described here to make its derivation transparent. First, a purposive search of peer-reviewed literature and authoritative policy and guidance documents was conducted in PubMed, Scopus and Google Scholar, together with the publications of the WHO and allied agencies, using combinations of terms relating to artificial intelligence, machine learning, maternal and child health, nursing, health equity, algorithmic bias, implementation science and low-resource settings. Sources were selected for relevance to four bodies of knowledge that the authors judged central to the problem: (i) health-AI ethics and governance; (ii) digital and algorithmic equity; (iii) respectful and humanised maternity care; and (iv) implementation science for health technologies. Second, recurrent failure modes and success conditions identified across these literatures were extracted and grouped thematically. Six themes recurred consistently and were judged to be both necessary and non-redundant: contextual validation, humanised relational care, community co-design, digital equity, data governance and longitudinal evaluation. Themes were prioritised on three criteria – frequency of appearance as a determinant of success or failure, specificity to AI (as distinct from general digital health), and salience to nurse-led practice in LMICs. Themes that were important but generic to all health programmes (for example, general financing) were retained as cross-cutting enabling conditions rather than as standalone domains.

The framework is intended to advance beyond existing guidance in three specific respects. Whereas the WHO guidance on the ethics and governance of AI for health provides high-level principles applicable across the health system (4), it is not operationalised for nurse-led community MCH practice or for the particular equity hazards of LMIC maternity care. Whereas implementation science models such as NASSS explain why health technologies succeed or fail organisationally (20), and implementation-outcome taxonomies specify what to measure (29), neither is specific to AI equity in MCH nursing. And whereas nursing-AI position statements articulate professional priorities (24), they do not integrate algorithmic-bias mitigation, digital-equity design and community co-design into a single nurse-led architecture. The contribution of the present framework is to bind these strands together around the nurse–patient relationship as the central mediator of safe and equitable AI integration, and to attach illustrative, equity-sensitive indicators to each domain. The framework is offered as a testable proposition rather than a validated instrument.

Three features distinguish this framework as a conceptual contribution rather than a reorganisation of established principles. First, it inverts the conventional logic in which equity is treated as a downstream outcome of technology adoption: here equity is repositioned as an upstream precondition, such that contextual validation and digital inclusion must be satisfied before, not after, deployment. On this logic an AI tool that has not been locally validated and made accessible is treated as not yet implementable rather than as implemented-but-imperfect. Second, the framework specifies a single, testable mediating mechanism – the nurse–patient relationship – as the pathway through which every domain is hypothesised to act on outcomes, rather than presenting equity, governance and humanised care as parallel and independent goods. This makes the framework falsifiable in a way that principle lists are not: it predicts that AI tools which strengthen relational continuity will improve engagement and outcomes, whereas tools that erode it will not, even where their technical performance is high. Third, it relocates governance and validation authority toward the community nurse and the served community rather than the developer or the central health system, reframing the nurse from an end-user of AI into a co-designer, gatekeeper and accountable interpreter of it. To our knowledge, no existing framework combines an equity-as-precondition logic, a single relational mediating mechanism, and nurse-and-community-held governance authority for AI in LMIC MCH care; it is this configuration, rather than any individual element, that constitutes the novel contribution.

### The six domains

**Figure 1:**
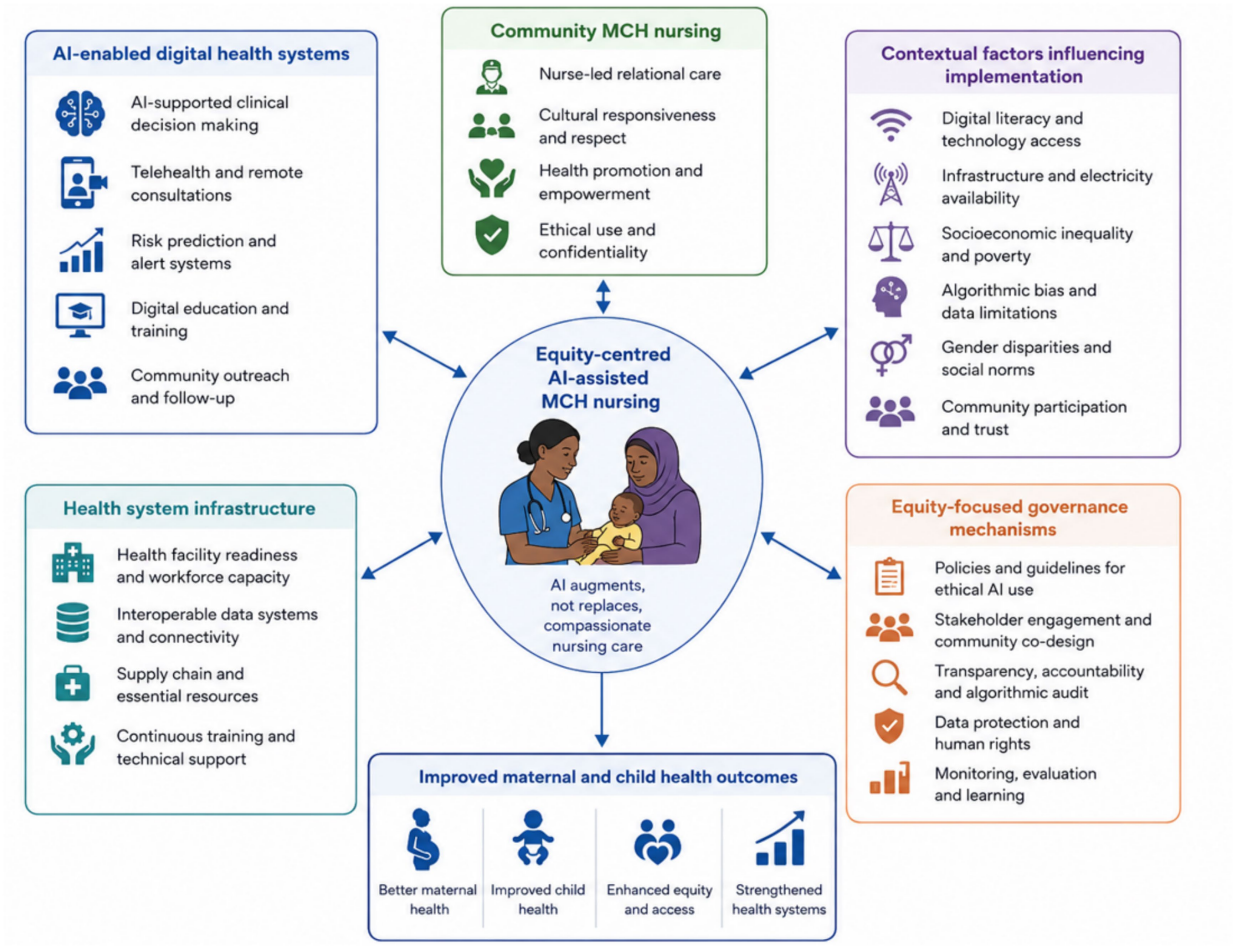
Conceptual framework for equity-centred, AI-assisted maternal and child health (MCH) nursing in low-resource settings. The diagram organises the framework around a nurse-led, equity-centred core and maps onto the six harmonised domains described in the text and Table 1: contextual validation of algorithms (algorithmic bias and data limitations; transparency and algorithmic audit), humanised and relational nursing care (the “Community MCH nursing” cluster: nurse-led relational care, cultural responsiveness, ethical use), community co-design and engagement (stakeholder engagement and community participation), digital equity and inclusion (digital literacy and technology access, infrastructure and electricity, gender disparities, and the health-system infrastructure cluster), transparent data governance (policies for ethical AI use, data protection and human rights), and longitudinal monitoring and evaluation (monitoring, evaluation and learning). The “improved maternal and child health outcomes” node represents hypothesised distal outcomes that are conditional on successful, equitable implementation rather than expected results of AI adoption. AI is presented as an augmenting tool embedded within broader health system strengthening, not as a replacement for compassionate nursing care.

The proposed framework (Figure 1; Table 1) integrates six interdependent domains. It is essential to acknowledge explicitly that the framework has not yet undergone empirical validation, stakeholder consultation, expert consensus or field testing in LMIC MCH settings. It represents a conceptual proposition grounded in the reviewed literature rather than a prescription for implementation, and its utility in practice requires prospective testing. The domains are not hierarchical but mutually reinforcing: deficits in any single domain can undermine the effectiveness and equity of AI integration across the others.

**Table 1:** Proposed framework for equity-centred, nurse-led ai-assisted maternal and child health nursing in low-resource settings (six harmonised domains).

Domain	Current limitation	Recommended approach	Scientific rationale	Measurable indicators (illustrative)
Contextual validation of algorithms	Many predictive models are developed on datasets from high-income settings and may not represent LMIC populations, reducing external validity; local validation data are rarely published.	AI models should be developed or recalibrated using locally representative datasets, with mandatory context-specific validation and bias auditing before deployment in any new setting.	Local validation reduces selection, measurement and deployment bias and supports safer model-informed decisions in diverse populations.	Proportion of deployed models with published LMIC validation data; documented bias audits; equity-stratified performance reporting.
Humanised and relational nursing care	Automated triage and model-driven prompts may erode relational continuity and respectful care if poorly integrated; nurses may have limited preparation in AI literacy and ethical use.	Embed AI tools within nurse-led relational care, respectful maternity care and trauma-informed practice; integrate AI literacy, algorithmic-bias awareness and digital ethics into pre-service and continuing education.	Trust, empathy and cultural safety shape maternal engagement, adherence and outcomes and cannot be reproduced by automated systems; prepared nurses interpret model outputs safely.	Patient-reported respectful-care measures; retention in antenatal and postnatal programmes; proportion of MCH nurses completing AI-literacy training.
Community co-design and engagement	Communities and service users are often insufficiently involved in designing digital interventions, reducing acceptability, cultural fit and sustainability.	Use participatory, culturally responsive and language-appropriate co-design; nurses should facilitate sustained community dialogue and feedback loops rather than token consultation.	Meaningful community participation improves acceptability, trust, relevance and the long-term sustainability of digital health programmes.	Proportion of programmes using formal co-design; community satisfaction and acceptability measures; documented adoption barriers.
Digital equity and inclusion	Access to devices, connectivity, electricity and digital literacy is unequal across gender, income, age and geography; women in LMICs may face gender-based restrictions on technology use.	Provide inclusive, low-cost, community-based access pathways (shared-device and community health worker intermediary models); prioritise reliable infrastructure and offline-capable platforms.	Equity-oriented access can prevent digital innovation from widening disparities; gender-transformative approaches are needed to address structural barriers.	Gender gap in digital access; proportion of women without device ownership; coverage of shared or proxy access models; facility connectivity.
Transparent data governance	Privacy protection, informed-consent procedures and accountability mechanisms are weak or inconsistently applied in many LMIC health systems.	Establish transparent ethical and regulatory frameworks for data protection, informed consent, algorithmic accountability and harm reporting, aligned with WHO principles for health AI.	Strong governance safeguards sensitive MCH data, strengthens public trust and is foundational to ethical and rights-respecting AI deployment.	Presence of national AI-health governance frameworks; proportion of programmes with documented consent processes; reported data-breach incidents.
Longitudinal monitoring and evaluation	Evidence on long-term effectiveness, cost, equity effects and sustainability is limited; most studies rely on short-term pilot designs.	Conduct longitudinal evaluation of clinical outcomes, implementation outcomes (adoption, fidelity, sustainability), costs, safety, equity effects and patient experience.	Long-term implementation-science evidence is necessary for responsible scale-up and policy adoption; equity indicators must be integral to evaluation.	Availability of longitudinal outcome data; inclusion of equity-stratified analyses; proportion of programmes with published implementation evaluations.

AI, artificial intelligence; MCH, maternal and child health; LMICs, low- and middle-income countries. The six domains correspond exactly to those described in the abstract, main text, Figure 1, Discussion, and Conclusion. AI here denotes machine-learning-based predictive and decision-support tools, not digital health interventions in general. The indicators are illustrative proposals to guide future evaluation; the framework is conceptual and unvalidated, and the relationships it implies between domains and outcomes are hypothesised pathways requiring prospective testing.

The first domain, contextual validation of algorithms, addresses the imperative that AI models be developed or recalibrated using locally representative datasets before deployment in any new LMIC setting. This domain connects directly to the reduction of selection, measurement and deployment bias, and to the generation of LMIC-relevant evidence on model performance and equity effects.

The second domain, humanised and relational nursing care, positions nurse-led compassionate practice, respectful maternity care principles and trauma-informed approaches as the non-negotiable foundation upon which AI tools are integrated. Because this foundation depends on a prepared workforce, the domain also encompasses nurse AI-literacy, ethical reasoning and algorithmic-bias awareness, ensuring that AI augments rather than displaces the interpersonal dimensions of care that are most predictive of MCH outcomes in low-trust, high-vulnerability settings.

The third domain, community co-design and engagement, operationalises participatory implementation through structured co-design processes involving nurses, community health workers, women of reproductive age and community stakeholders. Community co-design is the primary pathway through which cultural responsiveness, linguistic appropriateness and community trust are embedded into AI-assisted MCH programmes.

The fourth domain, digital equity and inclusion, addresses structural barriers to technology access across gender, income, age and geography, including the underlying infrastructure on which access depends. It requires that implementation actively maps exclusion risks, provides alternative and offline-capable access pathways, and adopts gender-transformative approaches to bridge the technology access gap.

The fifth domain, transparent data governance, establishes the ethical and regulatory infrastructure necessary for AI deployment to be trustworthy, accountable and rights-respecting. This domain aligns with the WHO principles of fairness, transparency, accountability, privacy and sustainability as applied to health AI (4, 27).

The sixth domain, longitudinal monitoring and evaluation, embeds implementation science methodology into AI-assisted MCH programmes. It requires evaluation of clinical outcomes, implementation outcomes (including adoption, fidelity, sustainability and equity effects) and patient-reported experience across programme life cycles, drawing on established implementation-outcome frameworks to operationalise accountability for both technical performance and equity impact (29).

The framework pathways are proposed as follows and should be read as hypothesised rather than demonstrated relationships. AI-enabled tools and health system infrastructure are inputs to a central, equity-centred and nurse-led MCH core; contextual factors and governance mechanisms are posited to shape implementation fidelity; and improved MCH outcomes—better maternal health, improved child health, enhanced equity and access, and strengthened health systems—represent the distal outcomes toward which the domains are intended to contribute, conditional on successful implementation. The proposed causal pathways are mediated primarily through nursing practice quality, community trust and health system responsiveness. Figure 1 presents the conceptual framework; Table 1 presents each of the six domains with its current limitation, recommended approach, scientific rationale and illustrative measurable indicators.

### Implementation pathways and interactions between domains

Although the six domains are interdependent, they are not interchangeable, and the framework is intended to be operational rather than merely descriptive. The domains stand in a definite relationship to one another, follow a logical implementation sequence, and connect to outcomes through a specifiable causal pathway. We set out each of these in turn and then illustrate them with a worked example.

#### Relationships between domains

The six domains can be grouped by function. Two are enabling preconditions that must be in place before an AI tool is allowed to influence care: contextual validation of algorithms (is the tool valid for this population?) and digital equity and inclusion (can the intended population actually access it without exclusion?). Two are integration conditions that govern how a validated, accessible tool enters practice: humanised and relational nursing care (the tool is embedded in, and subordinate to, the nurse–patient relationship) and community co-design and engagement (the tool reflects local priorities and language). Two are sustaining conditions that hold the system accountable over time: transparent data governance and longitudinal monitoring and evaluation. The dependencies run in a definite direction. Community co-design (a integration condition) shapes what contextual validation should optimise for, so the two are coupled; digital equity determines whether validation evidence generated in better-served sub-populations can be assumed to transfer to excluded ones; and data governance is a precondition for the monitoring domain, because equity-stratified evaluation cannot occur without trustworthy, consented data. A deficit in any enabling precondition is therefore not offset by strength elsewhere: an accurate but inaccessible tool, or an accessible but unvalidated one, should not proceed regardless of how strong the remaining domains are.

#### Sequence of implementation

These relationships imply a phased rather than simultaneous roll-out, summarised in Table 2. In a foundational phase, the enabling and governance scaffolding is established: data governance and consent processes are agreed with the community, exclusion risks are mapped, and the candidate algorithm is validated or recalibrated on locally representative data, with community co-design running in parallel so that validation targets and consent norms reflect local priorities. Only when these are met does an integration phase introduce the tool into nurse-led workflows, with nurse AI-literacy training, relational-care safeguards and clear rules that AI outputs are advisory inputs to nursing judgement. A consolidation phase then embeds longitudinal, equity-stratified monitoring, feedback loops and periodic recalibration, with explicit stop or roll-back criteria if equity-stratified performance deteriorates. The sequence is iterative rather than strictly linear: monitoring evidence feeds back to revalidation and re-design, and the community remains engaged across all phases rather than only at inception.

**Table 2:** Illustrative phased implementation pathway showing how the six domains are sequenced and gated.

Phase	Lead domains activated	Key actions	Gate to next phase (illustrative criteria)
1. Foundational (pre-deployment)	Contextual validation; digital equity and inclusion; transparent data governance; community co-design	Agree consent and data-governance arrangements with the community; map exclusion risks and establish alternative or shared-device access pathways; validate or recalibrate the algorithm on locally representative data and audit performance across equity strata; co-design what an AI output should trigger.	Locally validated model with equity-stratified performance audit; consent and governance processes in place; alternative access pathway available for excluded groups.
2. Integration (controlled introduction)	Humanised and relational nursing care; community co-design (continuing)	Train nurses in AI literacy, algorithmic-bias awareness and ethical use; embed the tool as an advisory input within nurse-led relational and trauma-informed care; establish that AI outputs prompt, but do not replace, clinical judgement; pilot in a limited area.	Demonstrated nurse competence and confidence; evidence that relational care and respectful-care measures are maintained; acceptable adoption and usability in the pilot.
3. Consolidation (scale and sustain)	Longitudinal monitoring and evaluation; transparent data governance (continuing)	Embed longitudinal, equity-stratified monitoring of clinical, implementation and patient-reported outcomes; run feedback loops to revalidation and re-design; schedule periodic recalibration and bias re-audit; apply explicit stop or roll-back criteria.	Sustained equity-stratified benefit including for the most disadvantaged groups; functioning audit and feedback mechanisms; pre-agreed roll-back triggers monitored.

AI, artificial intelligence; MCH, maternal and child health. The table illustrates the hypothesised sequence in which the six domains of Table 1 are activated; the phases are iterative rather than strictly linear, community engagement continues across all phases, and the gating criteria are illustrative proposals requiring empirical refinement. The pathway has not been validated and is offered to demonstrate how the framework could be applied in practice rather than as a fixed protocol.

#### Causal pathway to outcomes

The framework hypothesises a specific mechanism rather than a diffuse association between “good practice” and “better outcomes.” Proximally, the enabling and integration domains are posited to act on three mediators: nursing practice quality (more consistent risk stratification and referral, without loss of relational presence), community trust (willingness to engage, disclose and return), and health-system responsiveness (timely action on AI-supported referrals). These mediators, in turn, are hypothesised to influence intermediate outcomes earlier and more equitable identification of high-risk pregnancies, higher antenatal and postnatal retention, and more appropriate referrals which are the proximate drivers of the distal outcomes of reduced maternal and neonatal morbidity and mortality and narrowed equity gaps. Crucially, the pathway is conditional and can be broken: a technically accurate tool that bypasses the nurse–patient relationship is predicted to fail at the trust mediator and therefore not to improve outcomes, which is why humanised relational care is positioned as a mediating mechanism rather than as one optional good among others. The sustaining domains do not act directly on outcomes but protect the integrity of the pathway over time by detecting drift, bias and erosion of trust.

#### Illustrative application

Consider a district programme introducing an AI pre-eclampsia risk-stratification tool to support community nurses on home visits. In the foundational phase, nurses and women of reproductive age co-design the consent process and agree what a “high-risk” flag should trigger; the algorithm, originally trained on urban tertiary-hospital data, is recalibrated on district antenatal records and audited for performance across parity, age and distance-to-facility strata; and a shared-device pathway is established for women without personal phones. In the integration phase, nurses are trained to treat the risk flag as a prompt for fuller assessment and conversation rather than as a verdict, preserving the relational encounter that determines whether a woman accepts referral. In the consolidation phase, the programme tracks not only aggregate referral rates but equity-stratified retention and outcomes, and is contractually committed to suspending the tool if its benefit does not extend to the most distant and most disadvantaged women. This example shows how the domains operate together, in sequence, and through the relational mediator, rather than as a checklist of desirable attributes.

## Discussion

This Perspective has critically appraised the evidence base for AI-assisted MCH nursing, identified structural limitations in current implementation approaches and proposed a conceptual framework for equity-centred, nurse-led AI integration in low-resource settings. The central observation is that technological capability substantially outpaces the governance, workforce preparation, equity infrastructure and implementation science evidence required for responsible AI deployment in LMIC MCH contexts.

The evidence supporting AI applications in MCH is promising but incomplete, and it is important to keep AI-specific evidence distinct from the broader digital health literature. Most evaluations of AI predictive and decision-support tools are based on short-term pilot studies with limited evidence on sustainability, cost-effectiveness, equity effects and long-term clinical outcomes (13, 14, 20). Few studies assess whether AI systems improve care for the most vulnerable populations, and even fewer examine whether they inadvertently widen existing disparities. This gap is significant: interventions that demonstrate aggregate effectiveness may mask differential effects across income, geography, gender and ethnicity, with the most marginalised groups experiencing least benefit or greatest harm. The field requires longitudinal, community-based, equity-sensitive implementation research to generate the evidence necessary for responsible scale-up. It bears emphasis that the framework proposed here is a conceptual contribution grounded in literature synthesis, not an empirically validated model; specific caution is warranted against extrapolating from conceptual recommendations to implementation decisions ahead of prospective testing and stakeholder engagement.

These observations extend and nuance the existing literature in several respects. Earlier reviews of AI in LMICs identified infrastructure constraints and dataset representativeness as primary implementation barriers (12, 15). The present analysis adds that governance deficits, inadequate nurse workforce preparation, the resource intensity of sustained implementation, and the erosion of humanised care represent equally critical and less frequently addressed challenges. Compared with the implementation science literature on health-technology non-adoption and abandonment (20), the MCH AI field has paid insufficient attention to the sociocultural and relational dimensions of technology integration, particularly the nurse–patient relationship as a mediator of AI implementation outcomes. The distinct contribution of this framework, set out earlier, is not the assembly of these concerns but their configuration: equity treated as a precondition rather than an outcome, the nurse–patient relationship specified as the single mediating mechanism through which the domains act, and validation and governance authority located with the nurse and the served community. The phased pathway in Table 2 is intended to make this configuration operational by specifying the order in which domains are activated, the gates between phases and the points at which deployment should pause or roll back. The financial and workforce investments required to operationalise the framework’s six domains should be recognised as substantial rather than assumed to be cost-neutral additions to existing health system investments.

Policy implications follow, but should be read as conditional recommendations contingent on validation rather than as assured returns on AI adoption. National digital health strategies in LMICs should require mandatory local validation of AI models before MCH deployment, establish nurse-inclusive AI governance bodies, and integrate AI ethics and digital competency training into nursing curricula. International development partners and donors should fund equity-sensitive evaluations of AI-assisted MCH programmes rather than supporting proliferation of unvalidated tools, and should prioritise investments that address foundational health system infrastructure alongside AI applications. The WHO Global Strategy on Digital Health provides a policy anchor, emphasising that digital health investments be aligned with Universal Health Coverage commitments and prioritise underserved populations (28).

This Perspective has several limitations. As a Perspective based on a purposive, narrative review rather than a systematic review, it does not provide a comprehensive or quality-appraised synthesis of the evidence base, and its conclusions reflect a selected rather than exhaustive body of literature; the purposive search may not capture all relevant AI-assisted MCH initiatives, particularly those in grey literature, national reports or non-English publications. Readers should therefore weight the evidence claims accordingly. The framework is conceptual and has not been empirically validated, tested through stakeholder consultation or field-trialled; its proposed indicators, domain relationships and phased implementation pathway are theoretical propositions requiring prospective refinement. The LMIC examples cited are drawn from available peer-reviewed and programmatic literature and should be understood as illustrative of implementation contexts rather than robust evidence of effectiveness or scalability. The framework focuses primarily on community MCH nursing and may require adaptation for facility-based or specialist contexts.

The sustainability and scalability of AI-assisted MCH systems present additional challenges. Technologies that demonstrate feasibility in well-resourced pilot settings may fail to scale where implementation complexity is higher, governance weaker and resources scarcer. Governance structures must sustain accountability, community trust and equity orientation as programmes expand, with mechanisms for community feedback, algorithmic audit and responsive adaptation. The next steps required to advance this framework toward implementation readiness include empirical testing through prospective pilot studies in diverse LMIC contexts; stakeholder consultation with MCH nurses, community members, policymakers and technology developers; expert consensus to refine domain definitions and indicators; and iterative adaptation informed by implementation science feedback loops.

## Conclusion

Preventable maternal and neonatal mortality, childhood immunisation inequities and nursing workforce shortages in LMICs represent urgent and interconnected public health priorities. AI-assisted MCH nursing understood specifically as machine-learning-based predictive and decision-support tools has genuine potential to strengthen risk prediction, referral coordination and continuity of care in low-resource settings. However, this potential will only be realised if AI is implemented through frameworks that are equity-centred, nurse-led, contextually validated, culturally responsive and ethically governed.

The framework proposed in this Perspective advances a model of AI integration that preserves and reinforces humanised nursing care rather than displacing it. Its six interdependent domains contextual validation of algorithms, humanised and relational nursing care, community co-design and engagement, digital equity and inclusion, transparent data governance, and longitudinal monitoring and evaluation provide a structured architecture for responsible implementation grounded in implementation science, health equity theory and nursing ethics. Illustrative indicators for each domain create a starting point for accountability and evaluation. The framework is presented as a conceptual proposition for further development through empirical research, stakeholder consultation and field validation, rather than as a prescriptive model ready for immediate deployment, and its proposed links to improved outcomes remain hypotheses to be tested.

Three priorities for action follow, framed as conditional on the validation steps above. First, mandatory local validation of AI models before LMIC MCH deployment should become a regulatory standard rather than an optional aspiration. Second, nursing workforce development should integrate AI literacy, algorithmic-bias awareness and digital ethics as core competencies in both pre-service and continuing education. Third, implementation frameworks should be co-designed with communities, with particular attention to the inclusion of women who face the greatest risk of digital exclusion and algorithmic harm. When implemented responsibly and with unwavering commitment to equity, AI may augment the capacity of MCH nurses to reduce disparities, strengthen health systems and improve outcomes for women and children in the settings where need is greatest provided that the pace of deployment does not outrun the governance, equity and workforce foundations on which safe and equitable implementation depends.

## Data Availability

The original contributions presented in the study are included in the article/supplementary material, further inquiries can be directed to the corresponding author.
